# Geometrical vortex lattice pinning and melting in YBaCuO submicron bridges

**DOI:** 10.1038/srep38677

**Published:** 2016-12-23

**Authors:** G. P. Papari, A. Glatz, F. Carillo, D. Stornaiuolo, D. Massarotti, V. Rouco, L. Longobardi, F. Beltram, V. M. Vinokur, F. Tafuri

**Affiliations:** 1 Universitá degli Studi di Napoli Federico II, Dipartimento di Fisica, Napoli, I-80126, Italy; 2 Argonne National Laboratory, Materials Science Division, Lemont IL, 60439, USA; 3 Northern Illinois University, Department of Physics, DeKalb IL, 60115, USA; 4 NEST, CNR-INFM and Scuola Normale Superiore, Piazza San Silvestro 12, I-56127 Pisa, Italy; 5 Complesso Universitario di Monte Sant’Angelo, CNR-SPIN UOS Napoli, Napoli, 80126, Italy; 6 Seconda Universitá di Napoli, Dipartimento di Ingegneria Industriale e dell’Informazione, Napoli, 81031 Aversa (CE), Italy; 7 American Physical Society, Ridge NY, 11961, USA

## Abstract

Since the discovery of high-temperature superconductors (HTSs), most efforts of researchers have been focused on the fabrication of superconducting devices capable of immobilizing vortices, hence of operating at enhanced temperatures and magnetic fields. Recent findings that geometric restrictions may induce self-arresting hypervortices recovering the dissipation-free state at high fields and temperatures made superconducting strips a mainstream of superconductivity studies. Here we report on the geometrical melting of the vortex lattice in a wide YBCO submicron bridge preceded by magnetoresistance (MR) oscillations fingerprinting the underlying regular vortex structure. Combined magnetoresistance measurements and numerical simulations unambiguously relate the resistance oscillations to the penetration of vortex rows with intermediate geometrical pinning and uncover the details of geometrical melting. Our findings offer a reliable and reproducible pathway for controlling vortices in geometrically restricted nanodevices and introduce a novel technique of geometrical spectroscopy, inferring detailed information of the structure of the vortex system through a combined use of MR curves and large-scale simulations.

Superconductors are materials in which below the superconducting transition temperature, *T*_c_, electrons form so-called Cooper pairs, which are bosons, hence occupying the same lowest quantum state^1^. The wave function of this Cooper condensate has a fixed phase. Hence, by virtue of the uncertainty principle, the number of Cooper pairs in the condensate is undefined. As a result, the collective motion of the Cooper-pair condensate occurs without scattering (the scattering would have served a mean for counting Cooper pairs), i.e., without power dissipation^2^. This fundamental feature instrumental to technological applications of superconductivity is destroyed by magnetic vortices, tiny filaments of the magnetic field that penetrate the technologically important type-II superconductors. Vortices move under an applied current and destroy the global phase coherence leading to electric losses. Thus, immobilizing vortices, i.e., the problem of vortex pinning, is central to application-focused studies of superconductors^3^.

Superconducting bridges offer an irreplaceable laboratory for uncovering vortex properties. In an early work^4^ Parks and Moshel observed an oscillating magnetoresistance in what at the time were “extremely narrow” superconducting film strips of the order of 1 *μ*m wide and 100 nm thick in a perpendicular magnetic field, and justly guessed, well ahead of their time, that the oscillations were due to the sequential penetration of vortex rows into the strips. Now it is well established that if the strip is too narrow, vortices cannot enter the strip at all, but if the width is about a few coherence lengths, *ξ*, then vortices arrange themselves in a single and subsequently in distinct rows^5^,^6^,^7^. Moreover, if the strip is short enough, then an entry or an exit of even a single vortex might be visible in transport measurements^8^,^9^,^10^,^11^.

In narrow strips vortex behaviour is highly sensitive to the underlying interplay between geometrical restrictions and details of vortex arrangement^5^. A remarkable feature of the strips is that they allow to gain insight into the microscopic vortex behaviour combining numerical simulations of the vortex system and transport measurements^12^. Here we adopt the same integrated approach. Carrying out simultaneous numerical simulations, magnetotransport measurements, and visualizing the vortex penetration and dynamics in the strip, we calibrate the fingerprints of the magnetoresistance. This enables us to directly juxtapose the observed features of the magnetoresistance with the microscopic behaviours of the vortex system. We unambigously relate the resistance oscillations with the sequential penetration of vortex rows, separated by regions of geometrical pinning, and reveal the fingerprints of the magnetoresistance that evidence vortex lattice melting in the strip.

We carry out systematic transport measurements on high quality YBCO submicron bridges at both low and high magnetic fields. We observe oscillations of the magnetoresistance with a period corresponding to the penetration of additional vortex rows. We detect melting of the vortex lattice and identify the flow of the pinned vortex liquid controlled by plastic deformation of the vortex matter^13^.

## Experimental Techniques

**Figure 1:**
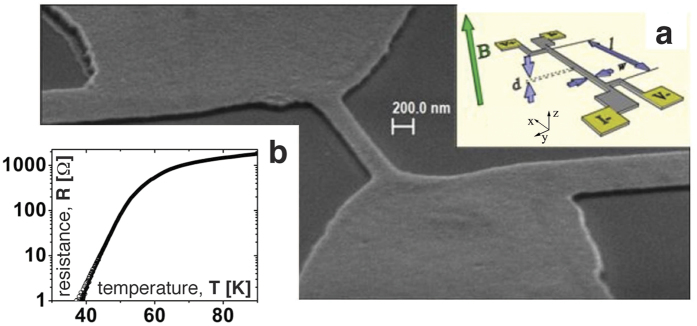
Sample setup S.E.M. image of the 230 nm wide sample. *Inset* (**a**) scheme of the magnetoresistance setup, *l* = 700 nm, *d* = 50 nm, *w* = 230 nm. *Inset* (**b**) Log-linear plot of the resistance, *R(T*) in the temperature range (30–90) K.

The sample fabrication is described in the Methods section. In order to explore the entire vortex phase diagram, we controllably degrade the samples. Varying parameters during each fabrication step of the ion milling procedure^14^,^15^,^16^ (i.e. the baking temperature of the photoresist, the duration of each single etching step and the interval between two of them, the cooling temperature of the sample during ion milling, and the power of the ion beam), we tune the oxygen desorption to achieve the desirable *T*_c_, *J*_c_ and *H*_c_^17^,^18^,^19^. Specifically, the oxygen desorption is tuned to have a transition to the superconducting state of about 50 K (see *R(T*) in Fig. 1, the transition temperature is defined by the middle of the drop in *R(T*)) to enable measurements in a wide range of temperatures (*T* ≥ *T*_*c*_). This allows to explore the vortex dynamics under the conditions ranging from the pinned to the flux flow state, yet remaining within a relatively small magnetic field range that is much smaller than the one in bulk samples (hundreds of Tesla). The dynamics of the vortex lattice and the vortex phase diagram remain unaltered, scaled to lower critical fields and temperatures^3^,^15^,^20^. Measurements are taken on the bridges with the width, *w*, lying in the interval 200 nm < *w* < 300 nm, their length being typically about 700 nm. Shown in Fig. 1 is a scanning electron microscope (SEM) image of an exemplary 230 nm wide sample. Finally, transport measurement are performed using a Helium-3 cryostat (HELIOX Oxford Instruments) equipped with different filter stages (for more details, see Methods).

## Experimental Results

**Figure 2:**
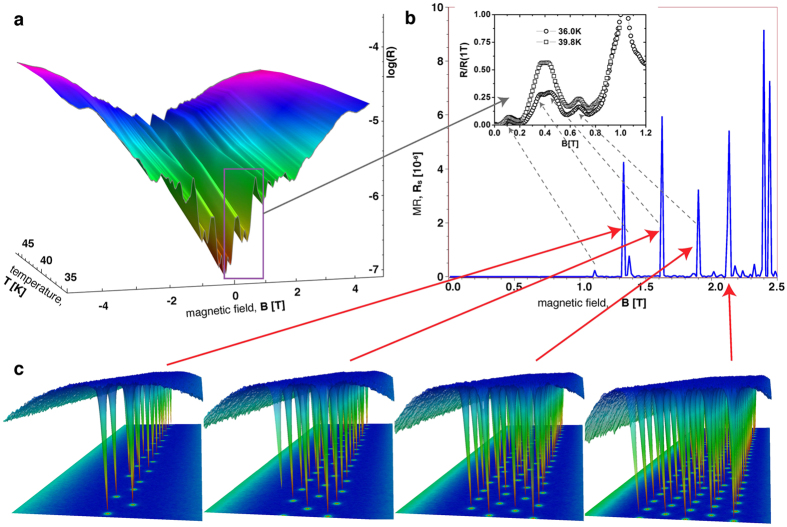
Magnetoresistance and vortex configurations. (**a**) Experimental MR of the 230 nm wide sample, in log-linear scale as a function of temperature. Important to note is that the position of the peaks does not change much with temperature. (**b**) Simulated MR of a 2D system with dimensions similar to those of the experimental system. The simulated temperature is lower than that in the experiment leading to sharper peaks, which are realized at higher fields. The periodicity and the relative peak heights are however reproduced correctly. The inset shows comparable experimental curves at 36 K and 39.8 K at low fields. (**c**) Vortex configurations at the peaks of the simulated MR curve. The peaks of the MR mark the appearance of a new vortex row entering the strip. The plots show isosurfaces of the superconducting order parameter amplitude and a color plot of the order parameter density as projection on the bottom.

Figure 2a shows a 3D surface plot of the differential magnetoresistance as a function of the temperature and magnetic field. One clearly sees the distinct peaks which do not change their position noticeably with variation of temperature. Shown in the inset of Fig. 2b are the representative data taken at two temperatures, 36.0 K and 39.8 K and moderate magnetic fields up to 1.2 T displaying the peculiar structure of the nonmonotonic behaviour of the MR. The peaks of the MR curves signal sequential penetration of the vortex rows into the strip^7^, as confirmed by numerical simulations (see below). The decreasing magnetoresistance immediately after the formation of the every next penetrating vortex row, reflects that with increasing field the vortex rows grow denser eventually merging into a central, nearly normal channel^7^. One further observes that peaks that correspond to the penetration of the second and third rows, nearly merge. Then there is a significant drop in the resistance before the fourth row enters. The relatively low peak that marks the appearance of the fourth row suggests that the four-row configuration experiences quite strong geometric pinning. The highest peak corresponds to the formation of the fifth vortex row, indicating that the five-rows configuration is unstable.

**Figure 3:**
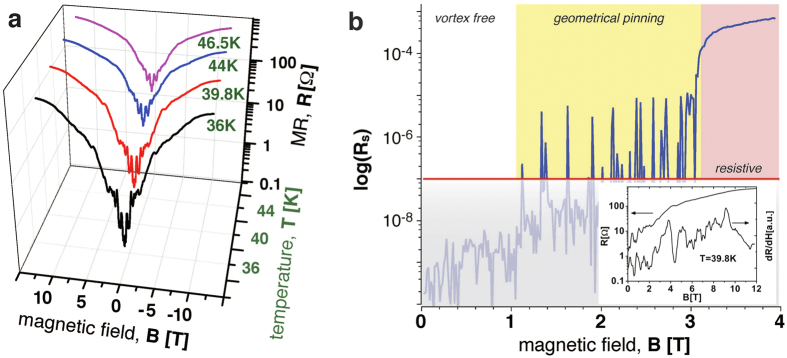
Full magnetic field range of experimental and simulated MR curves. (**a**) Measured MR curves at different temperatures in linear-log representation. (**b**) Simulated MR curves (linear-log plot). The red line indicates the limit of the numerical resolution. The inset shows a comparable MR curve obtained at 39.8 K along with its first derivative, which helps to detect MR oscillations at high fields.

The large field behaviour is illustrated in Fig. 3, where panel a) demonstrates the full range MR as a function of the magnetic field at representative temperatures. The character of the MR behaviour changes at about *B* = 3 T, as fully accounted by numerical simulations as shown in Fig. 3b, where the MR in log scale is reported as a function of the magnetic field. The plot log(*R*_*s*_) vs. *B* demonstrates a noticeable kink and change of the slope at 3 T (*R*_*s*_ denotes the dimensionless, simulated MR).

**Figure 4:**
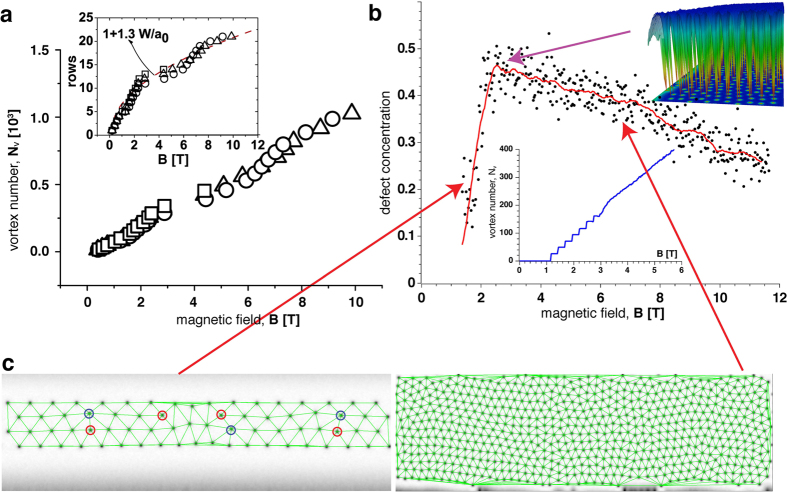
Vortex lattice melting transition. (**a**) The number of nucleated fluxons as a function of the applied magnetic field. The points shown by triangles, circles, and squares are recorded at 36.0 K, 39.8 K, and 46.5 K, respectively. The (red) dashed line is a linear fit of the data at 39.8 K. Inset: the number of the entered vortex rows as a function of *B*. The dashed curve is the function 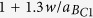 where 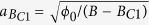 which represents the theoretical trend expected for an ordered vortex lattice. (**b**) Analysis of the simulated vortex lattices as a function of field. The plot shows the topological defect concentration (vortices with number of closest neighbours different from 6) obtained by Delaunay triangulation. The edge rows are not taken into account, such that the curve starts when the third row is formed. The scattering plot shows a typical run, and the red curve is an average of 12 runs with the different random initial configurations. When the vortex lattice starts moving at *B* ~ 3*T* the defect concentration shows a maximum (a typical vortex configuration at the maximum is shown at the top right corner). The inset shows the vortex number as a function of the magnetic field. At low fields, below the dynamic transition the vortex number shows a clear step-like behaviour when new vortex rows appear. At high fields the vortex number increases continuously. Two typical vortex lattice triangulations are shown in panel (**c**) (topological defects having 5 and 7 nearest neighbors are highlighted by red and blue circles, respectively, on the left.)

To gain an insight into the nature of this behaviour, we plot the number of vortices *N*_*v*_ as a function of the magnetic field in Fig. 4a. *N*_*v*_ is estimated as 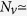
⌊1+*w*/*a*_0_⌋(*L*/*a*_0_), where *w* and *L* are the width and the length of the strip respectively, and 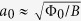 is the mean distance between vortices at *B* ≫ *B*_c1_ ∝ Φ_0_/*w*^2^, see refs 21,22. It is also assumed that *w* ≫ *ξ*, and ⌊*x*⌋ denotes the integer part of *x*. At 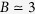 T the behavior of *N*_*v*_(*B*) changes to a continuous increase (which is easier seen in the simulations, Fig. 4, inset), where vortices do not enter as complete new rows, but rather individually. Furthermore, at about the same field there is a kink in the magnetic field dependence of the number of rows. The high-field part of the *N*_*r*_(*B*) dependence is well approximated by *N*_*r*_ = 1 + 1.3*w*/*a*_0_.

## Numerical Results and Discussion

To shed the light to the observed MR oscillations and gain insight into the behaviour of the system, we perform large-scale numerical simulations of a two-dimensional superconducting strip, with parameters of the experimental YBCO bridge, using the time-dependent Ginzburg-Landau equations^23^ (see Methods for technical details). Taking the zero-temperature coherence length of YBCO as *ξ*_0_ = 1.5 nm, we discretized the system in 1024 × 320 grid points having a physical size of 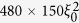 or 720 nm × 225 nm, close to that of the experimental system. A dimensionless current, measured in units of the depairing current, equaling to 0.05 (see Methods), is applied along the *x*-direction and a perpendicular magnetic field in *z*-direction is slowly increased from 0 to 0.1*B*_*c*2,0_. This field range spans the interval from 0 T to 14.5 T for the simulated system. At each field increment, the system is allowed to relax into a steady state and then the voltage along *x*-direction, proportional to the magneto-resistance (MR), i.e. *R*_*s*_ = ∂_*x*_*μ*, where *μ* is the scalar potential, is calculated. In YBCO the depairing current is ~300MA/cm^2^. Due to the method of measurement (see Methods), the way the magnetoresistance is obtained and its magnitude cannot be directly compared to the simulation.

To enable the observation of the MR oscillations, the simulations are performed at temperatures that are lower than those of the real experiment since the required averaging times for higher temperatures would have been prohibitively long. Yet, both the periodicity and relative peaks heights observed in the experiment are nicely reproduced in the simulations, see Fig. 2b. Note, the peak corresponding to the second vortex row has a smaller “side-peak”. This is due to the weak vortex interaction and the resulting slow formation of a hexagonally ordered double row. Therefore, it happens that the field is increased before the two rows are perfectly formed. At the simulated temperature, a full relaxation would take a very long time and becomes impractical. In fact, this instability of the second row can also explain the strong overlap of the second and third row peaks in the experiment. This overlap also causes those two peaks to apparently move closer to each other. The inset shows comparable experimental data and the corresponding simulated peaks are indicated by dashed arrows; note that because of the significantly higher temperatures in the experiment, the experimental peaks are much broader than their numerical counterparts and the first row can penetrate the experimental bridge at much lower fields as thermal fluctuations lead to a reduction of the surface barrier. The peaks in the MR mark the appearance of additional vortex rows with increasing field (see also Supplementary Movie). The mechanism behind the MR peak is that the formation of each additional row is the result of a second order phase transition between the different vortex states analogous to the well known transition at *B*_c1_ between the vortex-free and Shubnikov phases^7^,^5^. This implies that an increase of the MR due to suppression of the surface barrier for the vortex exit changes into a drop of the MR right after a new row emerges. The reason is that upon increasing the field, the newly formed vortex array gets denser, the regions with suppressed order parameter overlap more, thus the potential well for vortices in the inner part of the strip gets deeper. This deepening overweights the suppression of the surface barrier, hence the peak in the MR at the moment of the formation of the extra row^7^. Upon further increase of the field the suppression of the barrier becomes more important again, the growth of the MR wins again, and the process repeats itself. We stress here that since peaks in the MR correspond to well defined phase transitions in the vortex structure, these are the peaks of the MR (and not the minima) that have a clear physical meaning.

The vortex configurations at fields of the formation of new rows are shown in Fig. 2c as isosurfaces of the complex order parameter amplitude with a density projection at the bottoms. The behaviour of the simulated MR in the full range of the fields is presented in Fig. 3b as linear-log plots showing the transition to the resistive state at ~3*T*. The horizontal (red) line indicates the limit of the numerical resolution such that the shaded region below is not relevant. Above ~1*T*, in the yellow highlighted magnetic field range of the MR curve, vortex rows penetrate the system separated by field intervals in which the vortex lattice is geometrically arrested. At higher fields (highlighted in red) the system becomes resistive.

To further gain detailed knowledge about the nature of the structural transitions in the vortex system, we analyzed the evolution of the vortex configurations upon increasing the magnetic field by using Delaunay’s triangulation. The kink in the MR at 3 T shown in Fig. 4 strikingly resembles the manifestation of the so-called disorder-induced melting (DIM) predicted in refs 24,25. According to ref. 25 an increasing magnetic field enhances the destabilizing effect of the standard point-like disorder (e.g. oxygen vacancies in cuprates) on the vortex lattice. Thus at a certain magnetic field the topological defect-free, perfect vortex lattice transforms into a highly dislocated amorphous vortex configuration, where the vortex dynamics is governed by the motion of dislocations^26^. The DIM transition is characterized by the production of multiple dislocations. In contrast to disordered superconductors, in the strip the destabilizing role of pinning is taken by the edges of the strip, confining the vortex system and enforcing the symmetry different from the inherent symmetry of the vortex lattice and, therefore, generating stress in the vortex system near the edges. We conjecture that at some field *B*_CIM_ the *confinement-induced melting* (CIM) occurs in the vortex system. At this field, the generation of multiple topological defects (dislocations) starts near the edges, which together with the applied current forms dislocations and a new dynamic amorphous vortex phase. These dislocations are realized as a vacancy-interstitial pair propagating across the strip. The activated motion of vortices in this phase is controlled by the energy of the plastic deformation 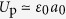, where *ε*_0_ is the energy of the vortex core and 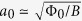 is the vortex lattice spacing. The dependence of the density of topological defects in the vortex lattice (i.e. the concentration of vortices with number of nearest neighbours other than six) on the magnetic field is shown in Fig. 4b. For the analysis, only vortices within rows having both neighboring rows on either side are taken into account. Therefore, the curve starts with the appearance of three rows since “edge” rows have no well defined number of neighbors in the used triangulation scheme. One sees that in the low field regime, for static vortex lattices, the number of defects increases. A maximum of defects is reached just before the system becomes resistive. In the dynamic state at higher fields the defect concentration decreases again due to dynamic annealing of defects in the inner part of the strip. The transition to the dynamic state is clearly seen in the number of vortices present in the system vs. magnetic field in the inset of Fig. 4b)], which is discrete at low fields with steps at the fields where additional rows appear in the system and continuous at high fields. Again, due to the lower temperature in the simulation, this effect is more pronounced than in the experiment, Fig. 4a. At this point we remark that disorder in form of e.g. inhomogeneities, grain boundaries, or surface defects can also influence the shape and location of the magnetoresistance peak. The experimental system is prepared from high quality films and shaped carefully. Furthermore, the results of our measurements indicate that there is no strong disorder which could play an important role, since our measurements were conducted in the resistive regime, where the applied current exceeds the depinning critical current. Weak pinning effects are not detectable since they need large spatial scales well exceeding the width of the strip in order to manifest themselves^3^. Additionally, the simulations of clean systems reproduce the main features – in particular, the envelope curve for the number of vortices follows the dependence expected in the clean state without surfaces. However, to check how disorder influences the simulations, we studied multiple scenarios of disorder by introducing various types of *δT*_*c*_ defects (i.e., spatial modulation of the critical temperature within the sample, meaning the linear coefficient, *ε*, in the TDGL equation): An amorphous structure and potential grain boundaries were modeled by weak random quenched noise, and Voronoi tessellation, tiling the domain with small polygons (of order of a few coherence lengths) with randomly chosen critical temperature close to the bulk *T*_*c*_. Other possible inhomogeneities or distortions of the surface, were simulated by weak localized defects. While all these defects can add slightly to the broadening and shift of the peaks, the effects of thermal noise — especially close to the experimental value — dominates the peak structure. In any case, the oscillation period of the magnetoresistance is still determined by the sample geometry only.

**Figure 5:**
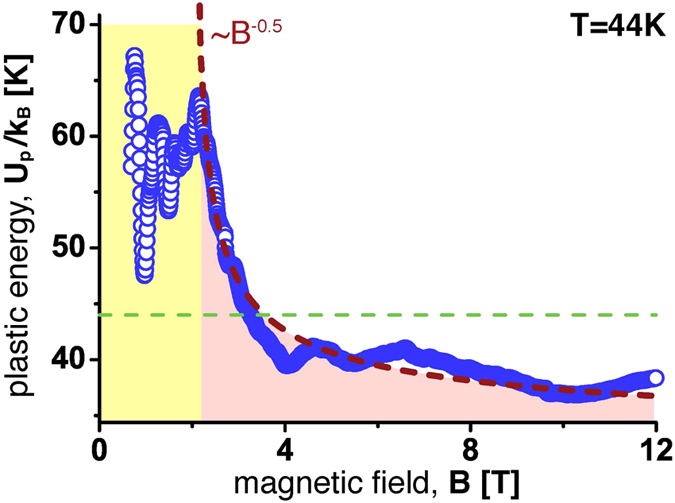
Plastic Energy *U*_*p*_ as a function of the magnetic field. The gray dashed line is a 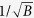 fit demonstrating that the data is consistent with the expected behaviour due to plastic deformation resulting from vortex shear^13^,^28^,^29^. The straight green dashed line corresponds to the temperature *T* = 44 K of the experiment.

Panel Fig. 4c shows snapshots of the vortex configuration with overlaid triangulation corresponding the low-field and high field amorphous phases, respectively. The field dependence of the activation energy *U*_p_ at *T* = 44 K is shown in Fig. 5. One sees that at *B* > *B*_CIM_ indeed 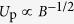 exactly as expected for vortex dynamics governed by the plastic deformations of the vortex system. Of course, the high field part of the curve where *U*_p_ becomes less than the temperature of the experiment should be taken with utmost reservation since the very concept of activated motion does not apply in this field range. However, in the proper field interval where *U*_p_ > *T*, the fit shows perfect agreement with the measurement.

## Conclusion

Our integrated experimental-numerical study of the vortex transport in the presence of a perpendicular magnetic field in a superconducting strip reveals the fingerprints of a low-field oscillatory magnetoresistance due to the peculiar interplay between the symmetry enforced by confinement or geometrical pinning and the inherent symmetry of vortex configuration corresponding to 2-, 3-, 4-, and 5-rows vortex phases. This opens an opportunity for a novel tool: characterization of the field-dependent vortex configuration through magnetresistance spectroscopy - analyzing measurements of the *R(B*) dependence together with large-scale simulations. At elevated fields we discovered a novel dynamic phase transition in the confined vortex system, confinement-induced melting, i.e., transition from the topologically perfect vortex configuration to an amorphous one saturated with dislocations. The oscillation period of the magnetoresistance of ~250 mT and the magnetic field at which the CIM occurs, are reproduced by the simulations.

## Methods

### Sample fabrication

The nanobridges are fabricated via processing a 50 nm thick YBCO film (in c-direction) covered by a 20 nm thick gold (Au) layer. Films are deposited by a thermal evaporation technique on yttrium stabilized zirconia substrates (YSZ) (Ceraco ceramic coating GmbH). The titanium (Ti) mask on the Au layer is prepared by the standard electron beam lithography and lift off procedures. Cold (−150 °C) ion milling is used to remove Au/YBCO from regions not protected by the Ti mask^16^. The Au top layer is finally removed by a further step of ion milling. Further details on the fabrication process are reported in ref. 14. Our measurements are performed on bare YBCO nanobridges (without Au cap layer), differently from what commonly done on YBCO nanowires which are always protected by Au to enhance as much as possible the critical current density.

### Measurements

Each type of filter is anchored at an independent temperature stage of the cryostat to work as low pass band filters with cutoff frequency *ν*_0_ < *ν*_*T*_ = *k*_B_*T*/*h*, which represents the thermal noise level where *k*_B_ and *h* are the Boltzmann and the Planck constants respectively. A handmade copper powder filter has been thermalized in the helium 3 pot of the cryostat, while *π*-filters have been anchored at the 1 K-pot. A further stage of commercial EMI filters has been used at 300 K, just after the voltage amplifier, to shield signals against electromagnetic environment. Current-voltage (*I–V*) curves are obtained via injecting an oscillating (about 7 Hz) current and acquiring an averaged (roughly 100 times) voltage curve through an oscilloscope (LeCroy WAVERUNNER 6100 A). Both the magnetoresistance and temperature, *R(T*), dependences, are taken employing a lock-in amplifier, feeding the sample with a current modulated at 17.3 Hz. Excitation signals were of the order of 300 nA to avoid heating and/or excessive perturbation of vortex dynamics^27^ effects. Magnetic field step was about Δ*B* = 10 mT, i.e., one tenth of the expected *B*_c1_ (see below). The setup for the zero bias *MR*’s is sketched in the Fig. 1(a).

### Simulation parameters

All simulations were performed using the time-dependent Ginzburg-Landau equations


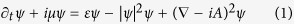



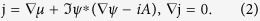


There equations were solved numerically by an implicit finite-difference method. Here *ψ* is the superconducting order parameter, *A* = (−*B*_*z*_,0,0)*y* is the vector potential corresponding to the applied magnetic field *B*_*z*_ perpendicular to the strip, j is the current density and *μ* is the scalar potential, *ε* = (*T*_*c*_ − *T*)/*T, T* is the temperature, *T*_*c*_ is the critical temperature. Note, that in this scaling, the zero-temperature coherence length *ξ*_0_ is the unit of length, the second critical field *B*_*c*2_(*T* = 0) the unit for the field, and the unit of *j* is 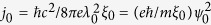, resulting in a depairing current density *j*_*dp*_ for **B** = 0 is given by 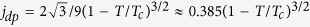.

The simulated strip is discretized by a square grid with resolution *ξ*_0_/2 – this is sufficient for the TDGL description and finer resolutions do neither change our results nor have any physical meaning, as microscopic theories are needed in that case (which are unsuitable to study vortex dynamics on larger length scales numerically). Thus for *ξ*_0_ ≈ 1.5 nm we need 1024 × 320 grid points to represent a system of physical size 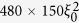 or 720 nm × 225 nm.

The simulated sample is periodic in the current direction (*x*) and superconductor/vacuum boundary conditions for the superconducting order parameter *ψ*, ∂_*y*_*ψ* = 0, are imposed at the surfaces in the shorter transverse direction, *y*. In order to collect sufficient statistics the results for the MR are averaged over 20 runs with the different random initial conditions.

For details on the algorithm and definition of all parameters, see ref. 23. The simulations were performed on Nvidia Tesla K20X GPUs.

## Additional Information

**How to cite this article**: Papari, G. P. *et al*. Geometrical vortex lattice pinning and melting in YBaCuO submicron bridges. *Sci. Rep.* **6**, 38677; doi: 10.1038/srep38677 (2016).

**Publisher's note:** Springer Nature remains neutral with regard to jurisdictional claims in published maps and institutional affiliations.

## Supplementary Material

Supplementary Information
