# Supplementary material for: Geometrical vortex lattice pinning and melting in YBaCuO submicron bridges
Source: Sci Rep. 2016 Dec 23;6:38677. doi: 10.1038/srep38677 (PMC5180090; doi:10.1038/srep38677)
Supplement: Supplementary Information [file srep38677-s1.pdf]

Supplementary Information: Geometrical vortex lattice pinning and melting in YBaCuO submicron bridges

G. P. Papari,[1] A. Glatz,[2, 3] F. Carillo,[4] D. Stornaiuolo,[1, 5] D. Massarotti,[6, 5]
V. Rouco,[1] L. Longobardi,[6, 7] F. Beltram,[4] V. M. Vinokur,[2] and F. Tafuri[6, 5]

[1]*Dipartimento di Fisica, Universitá degli Studi di Napoli Federico II, I-80126 Napoli, Italy*
[2]*Materials Science Division, Argonne National Laboratory, Argonne, Illinois 60439, USA.*
[3]*Department of Physics, Northern Illinois University, DeKalb, Illinois 60115, USA.*
[4]*NEST, CNR-NANO and Scuola Normale Superiore, Piazza San Silvestro 12, 56127 Pisa, Italy.*
[5]*CNR-SPIN UOS Napoli, Complesso Universitario di Monte Sant'Angelo, via Cinthia, 80126, Napoli, Italy.*
[6]*Dipartimento di Ingegneria Industriale e dell'Informazione, Seconda Universitá di Napoli, via Roma 29, 81031 Aversa (CE), Italy.*
[7]*American Physical Society, 1 Research Road, Ridge 11961 NY, USA.*

(Dated:)

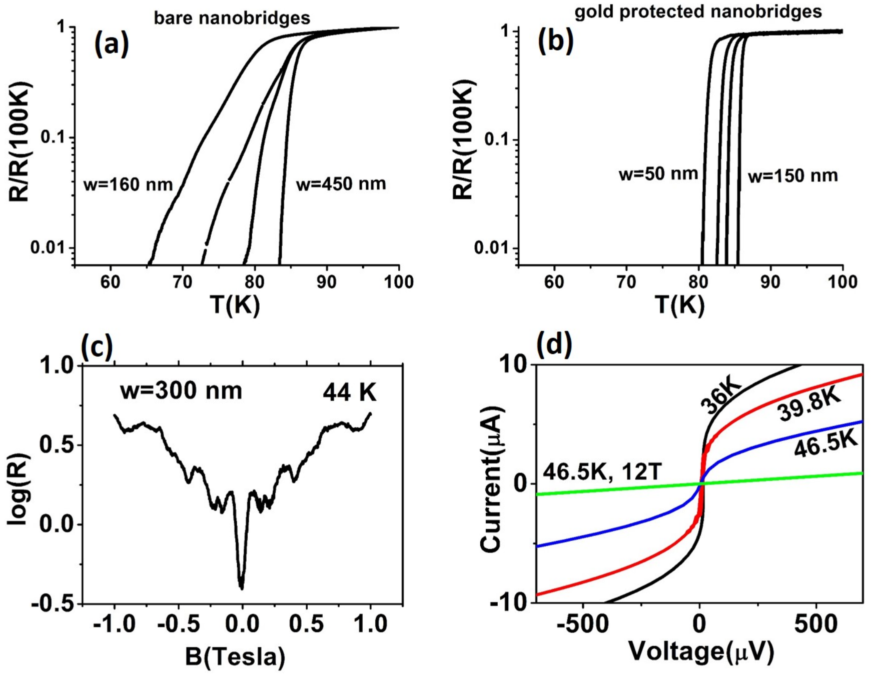

FIG. 1. (a) Normalized R(T)'s of bare YBCO nanobridges with different widths. (b) Normalized YBCO nanobridges protected with 50 nm of gold. (c) *MR* of a nanobridge about 300nm wide. (d) Current Voltage characteristics of the sample 230 nm wide at different temperature and fields. Black, red an blue curves have been acquired at different temperatures keeping off the external magnetic field, whereas the green curve has been recorded at 46.5 K with B=12 T.

I. DETAILS ON SAMPLE FABRICATION

During fabrication, the ion milling procedure consists of a finite steps process where the chamber is set with the following parameters: T= -140 ^{o}C, P(Ar)= $1.0x10^{-4}$ mTorr. Employing as beam current, beam voltage and accelerator voltage the values 5 mA, 315 V and 127 V respectively we get an YBCO etching rate of about 4 nm/min. In order to avoid harmful heating, the sample is etched with steps (gun-on) of 2 minutes, followed by 1 minute of gun-off. Getting larger step duration times we can to some extent tune oxygen desorption which regulates superconducting parameters as T_C, J_C and B_C. The dynamics of the vortex line lattice (VLL) and the vortex phase diagram remain unaltered, scaled to lower critical fields and temperatures [1, 2].

MR's shown here belong to samples etched with steps of 3 minutes resulting in reduced critical temperatures (T_C's) and critical currents (J_C's).

II. NANOBRIDGE CHARACTERIZATION IN PROXIMITY OF THE CRITICAL TEMPERATURE

High performances superconducting nanobridges can be fabricated leaving the gold protection on top of the samples [3]. In this case T_C's remain close to the unpatterned value (87 K) and J_C's are comparable with depairing supercurrent [3].

A comparison between bare and gold $R(T)$'s of samples processed with optimal milling duration time are reported in Fig.1(a) and (b) respectively. Bare samples display R(T)'s with lower T_C and a robust broadening which growths inversely to the sample width. Critical temperatures range from the unpatterned film value (87 K) when $w = 450\,nm$ to about $T_C = 65\,K$ for $w = 160\,nm$. Gold protected samples do not show any change in T_C from the unpatterned value down to $w = 150\,nm$ and the $50\,nm$ wide nanobridge has a $T_C = 80.5K$ larger than the boiling temperature of liquid nitrogen (77 K).

In Fig. 1(c) the MR of another nanobridge belonging to the same bunch of the one reported in the Letter is shown. The resistance oscillations are shifted at smaller fields according to the relationship $B_{C1} \propto \phi_0/w^2$[4]. In Fig.1(d) the current voltage characteristics (IV's) of the sample $230\,nm$ wide are reported. Temperatures of IV's are relative to the MR's reported in the Letter. The zero bias resistance is a sensitive channel to investigate vortex dynamics in superconducting nanosamples [1, 5]. The green curve has been acquired with $B = 12\,T$ when the sample is fully

resistive.

III. SUPPLEMENTARY MOVIE

The order parameter amplitude is visualized as function of the magnetic field – similar to Fig. 2c of the main text The final states of each magnetic field step are shown in the movies:

- HD resolution: https://youtu.be/5hJxtrg0wRE
- stereoscopic 3D, HD resolution: https://youtu.be/kJriIsv7Rh8

(these youtube movies are unlisted and therefore not publicly accessible.)

[1] F. Carillo, G. Papari, D. Stornaiuolo, D. Born, D. Montemurro, P. Pingue, F. Beltram, and F. Tafuri, Phys. Rev. B **81**, 054505 (2010).
[2] G. Papari, F. Carillo, D. Stornaiuolo, D. Massarotti, L. Longobardi, F. Beltram, and F. Tafuri, Physica C: Superconductivity and its Applications **506**, 188 (2014).
[3] G. Papari, F. Carillo, D. Stornaiuolo, L. Longobardi, F. Beltram, and F. Tafuri, Superconductor Science and Technology **25**, 035011 (2012).
[4] G. Stan, S. B. Field, and J. M. Martinis, Phys. Rev. Lett. **92**, 097003 (2004).
[5] I. Kokanoviè, A. Helzel, D. Babiè, C. Sürgers, and C. Strunk, Phys. Rev. B **77**, 172504 (2008).
